# Can we scale up a comprehensive school-based eye health programme in Zambia?

**DOI:** 10.1186/s12913-022-08350-2

**Published:** 2022-07-25

**Authors:** Ai Chee Yong, Anne Buglass, Godfrey Mwelwa, Ibrahim Abdallah, Ving Fai Chan

**Affiliations:** 1 Centre for Public Health, School of Medicine, Dentistry and Biological Sciences, Queen’s University Belfast, Northern Ireland, UK; 2 Vision Aid Overseas, Crawley, UK; 3 Vision Aid Overseas, Lusaka, Zambia; 4 College of Health Sciences, University KwaZulu Natal, Durban, South Africa

**Keywords:** School-based eye health programme, Scalability assessment, Uncorrected refractive error, Vision impairment, Childhood blindness

## Abstract

**Background:**

Globally, 19 million children have preventable vision impairment simply because refractive and eye health services are inaccessible to most of them. In Zambia, approximately 50,000 school children need spectacle provision. The School-based Eye Health Programme (SEHP) has been identified worldwide as a proven strategy to address childhood blindness. Given its great benefits, the Zambian government intends to scale up the programme. This scalability assessment aims to identify and evaluate the essential components of an effective SEHP, determine roles, assess existing capacities within user organisations, identify environmental facilitating and inhibiting factors, and estimate the minimum resources necessary for the scaling up and their proposed scale-up strategies.

**Methods:**

Five elements (innovation, user organisation, resource team, environment, and strategies for horizontal and vertical scaling-up) were assessed guided by the ExpandNet-WHO Nine Steps for Developing a Scaling-Up Strategy. Literature review on proven strategies to reduce childhood blindness and the credibility of SEHP implemented in resource-limited settings, document review on the pilot project, questionnaires, and stakeholders’ interviews were conducted to collect data for this assessment. Subsequently, twenty questions in the Worksheets for Developing a Scaling-up Strategy were used to report the assessment outcome systematically.

**Results:**

Additional components of SEHP incorporated in Zambia’s model enhanced the innovation’s credibility and relevance. The resource team was relatively competent in the pilot project, and the same team will be employed during the scaling-up. Potential change in political parties, the lack of supply chain, and unstable financial support were identified as inhibiting factors. The objectives of SEHP were aligned with the National Eye Health Strategic Plan 2017–2021, which supports the institutionalisation of the SEHP into the existing School Health and Nutrition Programme. For the pace of expansion, replicating SEHP to another district rather than a province will be more realistic.

**Conclusion:**

Scaling up a comprehensive SEHP in Zambia is feasible if sufficient funding is available. Additionally, the pace must be adapted to the local context to ensure that every component within the SEHP is intact.

**Supplementary Information:**

The online version contains supplementary material available at 10.1186/s12913-022-08350-2.

## Background

Globally, 19 million children have vision impairment (VI) mainly due to uncorrected refractive error (URE), congenital cataract and glaucoma, corneal opacities, trachoma, and retinal disorders [1]. URE is identified as the leading cause of VI, affecting 12.8 million children simply because they do not have access to refractive services and spectacle corrections. Unfortunately, approximately 90% of the heavy burden falls in low- and middle-income countries, which further stretches the health systems that were already under-resourced [2]. Because of poor vision, children were more likely to have a limited academic achievement that may affect their future career opportunities and livelihood [3–7] and experienced psychosocial effects such as depression, stress, and anxiety disorders which may negatively impact their quality of life [8–11].

While there is no published population-based information on the burden of childhood VI in Zambia, anecdotal evidence shows that an estimated 50,000 Zambian children had an unmet need for refractive services in 2020 [12]. To address this burden, [13] the Ministries of Health and General Education initiated a pilot comprehensive School-based Eye Health Programme (SEHP) collaboratively with Vision Aid Overseas, an eye care non-governmental organisation (NGO) focusing on reducing the burden of URE in low-resource settings. The pilot consisted of eye health screening, provision of free spectacle correction and prescription of eye ointment to treat common eye ailments such as allergic conjunctivitis, and dissemination of eye health knowledge to the community.

SEHP has been adopted as a successful platform to deliver interventions such as disseminating nutrition and health knowledge to parents to avoid Vitamin A deficiency blindness, accelerating vaccination uptake to avoid measles-related eye diseases, and promoting SAFE strategy to prevent trachoma (surgery for trichiasis, antibiotics, face cleanliness and environmental improvement). These innovative interventions reduced the number of blind children by 24% in the last 30 years, despite massive population growth [14].

Given the great benefits of SEHP, [15] the Zambian Ministries of Health (MOH) and General Education (MoGE) intend to scale up the SEHP to benefit more children. However, the scale-up must be done gradually and systematically to ensure the programme’s effectiveness and sustainability, besides providing children with quality eye health services. Therefore, this scalability assessment aims to identify and evaluate the essential components and operations of an effective SEHP, determine roles, assess existing capacities within user organisations, identify environmental facilitating and inhibiting factors for scaling up, estimate the minimum resources necessary for scaling up, and finally to propose strategies.

## Methods

### Assessment tools

The ExpandNet-WHO Nine Steps was used to guide the assessment for developing a scaling-up strategy for SEHP in Zambia [16]. It supported a systematic approach for institutionalising and expanding the comprehensive SEHP based on experience derived from the pilot project. We identified and assessed all the elements, including the innovation, user organisation, resource team, environment, and vertical and horizontal scaling-up strategies. (Table 1) We also explored these elements’ strengths and weaknesses, facilitating and inhibiting factors. Based on the findings, we made recommendations and proposed potential strategies for the scaling-up. Subsequently, the ExpandNet-WHO Checklist for assessing the potential scalability of pilot projects or other programmatic research [17] was completed to determine how much information or action is needed to facilitate a sustainable scaling up. The checklist was organised into 12 questions based on the elements outlined in the ExpandNet-WHO Nine Steps for Developing a Scaling Up Strategy [16].

**Table 1:** The elements for scaling up a comprehensive School-based Eye Health Programme

Elements for scaling up		Descriptions
Element 1	The innovation *The package of interventions or other new practices being scaled up.*	Comprehensive School-based Eye Health Programme (SEHP).
Element 2	User organisation/s *The institution or organisation intended to adopt and implement the innovation on a large scale.*	Zambian Ministry of General Education (MoGE) and Ministry of Health (MOH), and Vision Aid Overseas (VAO).
Element 3	Resource team *The individuals and organisations promote scale up of the innovation.*	VAO country team, MOH’s and MoGE’s representatives, Provincial Health Director, Provincial Education Officer, District Health Officer, District Education Board Secretary, School Heads/ Head Teachers, Schoolteachers, Ophthalmic Nurses, Ophthalmic Clinical Officers, Ophthalmologists, Optometry Technologists, focal person for mobile eye health clinics.
Element 4	Environment *The conditions and institutions are external to the user organisation but affect the success of scaling up.*	Macro- and micro-environment, which considers the cultural views and receptivity of the local community towards the SEHP, and the political support to incorporate child eye health into the national health policies.
Element 5	Scaling-up strategies *The scaling-up strategy is the plan for promoting, expanding and institutionalising the innovation. Development of an effective scaling up strategy assesses the elements of scaling up and the actions needed to improve them and then examines the strategic choices for each of the four types of scaling up.*	Types of scaling up: a). **Horizontal scaling up** refers to the expansion or replication of the pilot to different geographical areas or serving different populations. b). **Vertical scaling up** refers to institutionalising the programme embedded in the policies, organisational structures, and operational guidelines.

### Key informants

Individuals and the representative of the institutions who were involved and engaged in the pilot project were identified as key informants for the assessment. The key informants included the representative of the Zambian Ministry of Helath and Ministry of Genereal Education, local project coordinator and director, and Vision Aid Overseas’ Programmes Director and the Deputy Programmes Director.

### Data collection

A global health optometrist (ACY) engaged in several eye health projects in low resource settings conducted the scalability assessment from September to December 2020, employing a mixed-methods approach. ACY reviewed the literature on proven strategies in reducing the burden of childhood VI, the credibility of SEHP, which is implemented in both high-income and low- and middle-income countries, and the effectiveness of a comprehensive SEHP in improving health and educational achievement. ACY also reviewed the Standard Guideline for a Comprehensive SEHP developed by the International Agency for the Prevention of Blindness, [13] project reports from Vision Aid Overseas, the Zambian National Health Strategic Plan 2017–2021, [18] National Eye Health Strategic Plan 2017–2021, [19] and the National School Screening Programme Protocol 2020.

After the literature and reports’ review, a set of questionnaires exploring the five assessment elements was developed. (Online Supplementary File 1.) Based on the experience gained from the recent pilot project, the local stakeholders completed the questionnaire. Due to the COVID-19 pandemic, interviews with the key informants were conducted via an online platform and phone call. A semi-structured guide was developed to collect information relating to stakeholders’ views on consensus and partnership, integration of SEHP into the existing School Health and Nutrition Programme (SHNP), and the health information system in Zambia. (Online Supplementary File 2.) Because the interview was not recorded, after inserting key informants’ inputs according to the interview questions in a Word document, the interviewees were asked to re-check the content and make amendments accordingly to ensure data accuracy. Given the small sample size and to protect the confidentiality of the identity, we utilised “QR” when reporting the findings from the questionnaires, “KII” for key informant interviews, and “DLR” for document and literature review.

### Data management and synthesis

The Worksheets for Developing a Scaling-up Strategy provided a detailed set of questions for the strategic planning process [20]. We tabulated the findings from the three different sources in a table, where we grouped the findings into themes. The themes included facilitating and inhibiting factors at the micro- and macro-level, lessons learnt from the pilot project, capacity within the resources teams, potential to include additional local key stakeholders during the scaling-up and the approaches, and views on strategies to scale up the SEHP horizontally and vertically. We triangulated the data by comparing the responses from the questionnaires, the outcomes of the interviews, and extraction from literature and document reviews within the themes to determine the convergence, complementarity, and divergence of information.

## Results

### Element 1 - an assessment of the components of the piloted comprehensive School-based Eye Health Programme

The comprehensive SEHP piloted in the Kafue district has demonstrated remarkable achievement in the Zambian context (from 17th June 2019 to 17th December 2019). The pilot screened 18,713 Grade 1 to 9 children (coverage of 43.15%) from the 73 schools and identified 3817 children with eye problems (dispensed 600 pairs of spectacles, referred 68 children to a tertiary hospital for further care, and prescribed more than 3000 bottles of eye drops). Out of all the screened children, 33.9% were aged 14 and older, 29.7% were 11 to 13 years, and 36.4% were younger than 10 years old. (Table 2) The most common ocular conditions were allergic conjunctivitis (80.5%) and uncorrected refractive error (16.7%). (Table 3) A total of 621 children were prescribed spectacles, and 67.0% of them were short-sighted.

**Table 2:** Demographic characteristics of screened children (*n* = 18,713)

Demographic Characteristics	Number of Children, n (%)
Sex	
Boy	8608 (46.0%)
Girl	10,105 (54.0%)
Age groups	
≤ 7 years	1661 (8.88%)
8 to 10 years	5150 (27.5%)
11 to 13 years	5560 (29.7%)
≥ 14 years	6342 (33.9%)
**TOTAL**	**18,713 (100%)**

**Table 3:** Diagnosis of ocular disorders in screened children (*n* = 3817)

Type of Ocular Disorders	Number of Children, n (%)
Uncorrected Refractive Error	621 (16.7%)
• Myopia	• 416 (67.0%)
• Hyperopia	• 80 (12.9%)
• Astigmatism	• 125 (20.1%)
Allergic Conjunctivitis	3073 (80.5%)
Cataract	23 (0.60%)
Corneal Scars	14 (0.37%)
Amblyopia	8 (0.21%)
Strabismus	15 (0.39%)
Retinal Disorders	2 (0.05%)
Others	61 (1.60%)
**TOTAL**	**3817 (100%)**

To address the shortage of human resources, this pilot project employed a strategy to train the schoolteachers as vision and eye health screeners. *“Medical personnel were not able to screen over 18,000 learners. The training of teachers helped build this cadre’s skill to conduct the initial screening that brought the number to be screened by medical staff to reasonable levels.”(QR 1)* With this effort, a total of 154 school teachers were trained to detect common eye problems and make referrals to secondary or tertiary levels of care. Besides, the project team also engaged with three local optometry technologists (OTs) and one local ophthalmologist and utilised three ophthalmic clinical officers (OCOs) and ophthalmic nurses (ONs) to conduct the mobile eye health clinics (MEHCs) in 6 zones within the Kafue district.

Many school vision screening projects conducted in Zambia were mainly event-based (routine practice), lacking consistency and scale. NGOs heavily drove the project, and as a result, the establishment of local ownership and leadership was limited. Table 4 demonstrated the advantages of the Zambia model that was piloted over other routine practices using the 3-point scale. A few prominent components in the pilot Zambian model were (i) the inclusion of local community engagement, (ii) programme monitoring and evaluation, and (iii) building a vision for up-scaling the programme. The local stakeholder reflected and stated, “*engagement with local authorities and communities are the entry points to any community, without which no community-based programme can succeed.”(QR 2).*

**Table 4:** Comparison of components between comprehensive School-based Eye Health Programme and routine practices

Components	Comprehensive SEHP (Zambia’s pilot model)^a^	Routine practices
Training local resources		
• schoolteachers and eye health personnel	++	+
Screening		
• visual acuity	++	++
• eye diseases (allergic conjunctivitis, cataract, glaucoma, cornea opacities)	++	+
Referral		
• to secondary/tertiary hospitals	++	+
• provide transportation and meal subsidy	++	–
Treatment		
• prescribe spectacles and provide eye medications – free of charge	++	+
Health promotion		
• among schoolteachers	++	++
• among parents	++	+
• community leaders and members	++	–
Sensitisation of the local community		
• community leaders and members	++	–
Monitoring and evaluation		
• spectacles wearing rate	++	+
• experience and perception - focus group discussion	++	–
Upscale	++	–

“-”: rarely practise; “+”: partially practise; “++”: fully practise

^a^Source of information: Report from the pilot project, and questionnaires

A SEHP protocol that the MOH, MoGE and VAO developed included a comprehensive care package consisting of crucial components in improving child eye health by enhancing services uptake and awareness. Table 5 details the descriptions of components applied in different settings.

**Table 5:** Description of components of the comprehensive School-based Eye Health Programme

Components	Settings	Descriptions
Training of schoolteachers	Workshops (conducted by OCOs, ONs and OTs)	• A two-day training (both theory and practical) will cover common eye diseases in children, refractive errors, general eye health, hygiene and face washing, eye screening and recording, and identifying children with poor vision in the classroom child protection and safeguarding.(DLR)
Screening	Schools (conducted by schoolteachers)	• Visual acuity measurement. • External eye observation (cornea, eyelid, pupil, conjunctiva, sclera). (DLR)
Referral	MEHCs (conducted by OCOs, ONs and OTs)	• Examinations of referred children who failed screening at schools which including: ➢ to perform cycloplegic refraction and prescribe spectacles if significant refractive error was observed. ➢ to perform a detailed eye examination and prescribe eye medications when necessary. ➢ to refer children with complicated eye conditions to hospitals. (DLR)
Treatment	Secondary/ Tertiary hospitals (examined by ophthalmologists, optometrists/OTs)	• Manage complex eye diseases which may require surgical interventions, e.g. cataract, glaucoma, or more complex refraction. (DLR)
Service delivery	Schools, MEHCs	• Prescribe spectacles (ready-to-clip spectacles) and eye medications onsite or deliver to schools (custom-made spectacles). • Provide per diems to referred children’s families to cover transportation expenses and meals. (DLR)
Monitoring and evaluation	(i) Schools, MEHCs, Hospitals (by teachers, OCOs, ONs, ophthalmologists)	• Schools, MEHCs and hospitals work together to have a proper data monitoring system to follow up on those referred children who attended the MEHCs/hospitals. (QR 1)
	(ii) Schools (by teachers, MOH, MoGE)	• Evaluation of spectacles wearing rate. • Focus group discussion on perceptions towards SEHP and interventions such as spectacles and eye medications. (QR 1)
Health promotion and education	Schools, communities (conducted by schoolteachers)	• Workshops (Parent-Teacher Association platform) for parents and local leaders cover topics such as the importance and stigma of spectacles wear, eye health hygiene, face washing (to prevent trachoma and eye infections), and proper eye health nutrition (Vitamin A), and promote health-seeking behaviour. (DLR, KII 1)
Engagement with local authorities and communities	Schools, communities (conducted by VAO, accompanied by District Health Officer and District Education Board Secretary)	• Visit schools and inform School Heads about the programme and seek support. • Visit communities and meet local leaders to inform them about the programme and seek support. (DLR, QR 2)

*OCOs* Ophthalmic clinical officers, *ONs* Ophthalmic nurses, *OTs* Optometry technologists, *MEHCs* Mobile eye health clinics, *DLR* Document or literature review, *QR* Questionnaires, *KII* Key informant interview

The review of the available policies showed that the SEHP aligns with the National Health Plan 2017–2021, the National Eye Health Strategic Plan 2017–2021, and VAO’s mission. In the pilot, all the referred children attended the MEHCs (*n* = 2818, no loss-to-follow-up), and an additional 3140 community children turned up in MEHCs seeking eye examinations. Reflecting upon this, we assume that the local communities’ response seems promising, and they showed support for the programme.

### Element 2 - an assessment of user organisations (Vision Aid Overseas, the Zambian Ministry of Health, and Ministry of General Education)

All three organisations (VAO, MOH and MoGE) were involved fully in the Kafue district’s pilot project. VAO took the lead on supervision and coordination while the National Eye Care Coordinator (representative of the MOH) and District Education Board Secretary (representative of the MoGE) closely supported VAO. The collaboration relationship and future partnership for the scaling-up among the three organisations were clear when interviewing the local stakeholders, “*The existing roles and collaboration among them are fine” (KII 1).* However, VAO, the lead institution for the pilot project, suggested that *“Ultimately, MoGE will be the owner for the SEHP. Therefore, a Memorandum of Understanding between the Ministries should be signed to ensure that each party plays its role accordingly during the scaling-up”(QR 1).*

Organisations’ competency in various sectors, namely leadership, training capacity, technical skill, project implementation, financial resources, and human resources, were explored. Our document review showed that MOH could effectively train schoolteachers as screeners, while local human resources such as OCOs, ONs and OTs can be mobilised to districts where health personnel are limited. Four local institutions can train eye health personnel: Levy Mwanawasa Medical University, University of Zambia, Kitwe Teaching Hospital, and Ndola Teaching Hospital. Triangulation of questionnaires and interview findings suggested that the leading team will need to be further equipped with managerial skills, capacity for advocating and researching, and monitoring and evaluation skills.

Due to the lack of reliable supply chains in Zambia for affordable quality spectacles, VAO provided children with spectacles and eye medications during the pilot project – *“VAO provided all supplies, spectacles and medications.”(QR 1)* To address this challenge, working with the African Eye Institute or a regional supply chain was suggested to reduce the cost of spectacles and eye medications. In terms of physical facilities, schools were used as screening venues while MEHCs as treatment points.

The most significant inhibiting factor is that no policy and legal framework included SEHP, despite its effectiveness in targeting child eye health disorders and other cross-cutting issues such as Vitamin A deficiency. *“The legal framework is not yet in place to support the implementation of the comprehensive SEHP. That’s the reason why we are working hard on developing a protocol for the programme, and test the pilot on the ground” (KII 3).* The availability of financial support was identified as another inhibiting factor highlighted by the local stakeholders. Furthermore, VAO, an NGO that heavily relies on donors and grants, is uncertain of the available funding for the scaling-up.

### Element 3 – an assessment of the environment for scaling up

#### Micro-environment - factors such as interaction among people and organisations and logistic accessibility

The pilot project evaluation revealed that the schools and communities showed no objections to government initiatives as school heads and community leaders felt respected when informed about the SEHP plan. Schoolteachers and health personnel were dedicated to participating in screening and management programmes for children because the organising committee created an enabling environment to support teachers with training, small incentives and meals, and transportation reimbursement. Furthermore, *“Considering that the MEHCs were set up in schools, they were accessible by all people.”(QR 2).*

One prominent challenge was highlighted: *“Myths and misconception and ignorance on eye health” (QR 1)* were common among the community and the local authorities. With this, the pilot project incorporating components such as community sensitisation is crucial to enhance eye health awareness and, therefore, increase service uptake from the locals.

#### Macro-environment - factors that may have a greater impact on the scale-up effort, such as at the political, economic and societal level

VAO has a well-established partnership with the Zambian MOH and MoGE. The implementation and advocacy of comprehensive SEHP may be affected if there is a change in existing government administration. However, the political factor is beyond the team’s control. According to the local stakeholders, the time it takes for the government to include SEHP into the existing SHNP was uncertain. They commented, “*Really very difficult to tell. Some proposed programmes may take a shorter time (within a few weeks!); others may take a longer time for approval. It really can’t (be) predict(ed). It involved politics, political will, and funds eligibility.”(KII 1, KII 2)* Besides, other health initiatives may also compete with SEHP.

The supply chain has yet to be developed and hence, inadequate in Zambia. Currently, there are only two suppliers of lenses and frames. VAO is responsible to source for frames, lenses and medications to support the management of referred children. VAO has applied to the Zambia Revenue Authority for Public Benefit Organisation status and envisaged that tax exemption would be granted to minimise the costs.

### Element 4 – an assessment of the resource team for scaling up

The scaling-up of the SEHP requires a team working from the top at the national level to the province and district level, and finally to schools and communities. *“The situation is that we have already got the support from the national level, and now we can work with the ground – the district level planner. The national level has to authorise first before we can work with the district level.”(KII 2)* The leading team which developed and tested the pilot project will be readily available during the scaling-up. The assessment through questionnaires and interviews demonstrated that they possess capacities such as:

- in-depth understanding of the user organisation’s capacities and limitations;
- capacity to train members of the user organisation;
- effective and motivated leaders with a unifying vision who have authority and credibility with the user organisation;
- understanding of the political, social and cultural environments within which scaling-up takes place;
- ability to advocate in favour of the innovation with policy-makers, government officials and programme managers;
- skills and experience with scaling-up;
- availability to provide support over a multi-year period.

Nevertheless, continuous effort to strengthen the capacities within the leading team is needed.

One highlight was that the Kafue pilot project had built the competency within the user organisations to sensitise the local community, train many schoolteachers, and coordinate logistics. Besides, information such as the prevalence of children needing spectacles or eye drops helps effective future planning and consumable procurement. Given the size of the resource team, there is a need to recruit several project officers to oversee the project at the simultaneous implementations in multiple districts. Besides, the interview with the local stakeholder highlighted that “*Senior Medical Superintendents of Eye Hospitals/Clinics and District Education Board Secretary offices will be an important resource during the scale-up.” (KII 3).*

### Element 5a – determining the role of policy/legal/political scaling up (vertical scaling up)

The National Health Strategic Plan 2017–2021 (NHSP) has included eye health as a non-communicable disease that needs to be prioritised [18]. The 3rd National Eye Health Strategic Plan (NEHSP) aims to achieve eye health coverage across the country to at least 90% by 2021 [21]. Furthermore, SEHP will likely cover the maximum number of children and be a practical approach to achieving this goal.(DLR).

Despite the strategy of capacity building among schoolteachers and community leaders to detect common eye diseases as mentioned in NHSP 2017–2021, a description of the comprehensive SEHP was not highlighted nor emphasised. The outcome evaluation of the pilot project has shown that SEHP has excellent potential to contribute to a few critical objectives stated in NEHSP 2017–2021.(Table 6) Therefore, a policy change is considerably needed.

**Table 6:** Objectives and strategies in the 3rd National Eye Health Strategic Plan [19]

Objectives	Strategies	Will the comprehensive School-based Eye Health Programme contribute to this?^a^
4.0 Eye health system strengthening	4.1.1 To promote good eye health and prevention of eye diseases by 100%	√
	4.2.1.2 To reduce the prevalence of active trachoma by 50% in children 1–9 years old	√
	4.2.3.1 To provide refractive services in more than 50% of the districts in the country	√
5.0 Integration with the wider health system	5.1.1 To ensure non-eye health workers have a better understanding of eye health conditions and can take appropriate actions	√
	5.2.1 To increase the number of eye health referrals that receive treatment in eye health facilities by 20%	√
	5.3.1 Children with eye conditions are identified through a school vision screening programme	√
6.0 Equity of access to eye health services	6.1.1 People in rural areas access basic eye health services in district hospitals	√
7.0 Strong and effective partnerships	7.1 To enhance the partnership between government, private institutions and co-operating partners in the eye health sector	√
8.0 Research and evidence	8.1 To generate evidence-based data for eye health specific to the Zambian context	√
9.0 Monitoring and evaluation	9.1 To strengthen the monitoring and evaluation in the delivery of the NEHSP 2017–2021	√

^a^Source of information: Report from the pilot project and questionnaires

The government’s full support ensures SEHP’s sustainability, referring to resource allocation such as human resources (eye health personnel in public hospitals) and funding (national health budget). Therefore, the integration of SEHP into the SHNP is critical if the government includes eye health as one of the components of SHNP. One strategy to achieving this is through advocacy to the government (i.e. vertical upscaling).

There are a few valid reasons that the inclusion of child eye health into SHNP will be an effective approach to address the burden of childhood blindness: (i) SHNP is already established in all the schools in Zambia, and therefore implementation will be made easier; (ii) besides nutrition and other health-related issues, eye health is vital in children’s cognitive and psychosocial development; (iii) the integration of the eye health into SHNP was shown to be more cost-effective as compared to the individual programme [22].

VAO has committed initial seed funding based on the available grants from international donors. Longer-term funding would need to be secured for national scale-up and may be done through bringing more stakeholders on board and/or the government securing bi-lateral funding for the programme. Due to the SEHP not being included in any public legal policies, there will be limited government support. Seeing this challenge, vertical upscaling plays a significant role in institutionalising the programme so that the MoGE can allocate that budget through the SHNP.

The inclusion of SEHP into the existing SHNP ensures sufficient financial resources to support the large-scale expansion. With this vision, the process of institutionalising the programme started in 2016. Resulting from the close collaboration among VAO, MOH and MoGE, a National School Vision Screening Protocol was developed. Besides, the government agencies’ involvement in designing and testing the Kafue district’s pilot project can help achieve this goal.

During the pilot project, VAO has supported all the expenses, including training and allowances for schoolteachers, consultancy fees for experts, spectacles and medications supplied to children, community sensitisation, mobile eye clinics’ incurred expenses (fuel, drivers, and vehicle maintenances), and allowances for the public sector eye health team (OCOs, ONs and ophthalmologist) during the activities. The MOH covered the public sector eye health team’s salary.

### Element 5b –determining the role of expansion scaling up (horizontal scaling up)

In Zambia, there are 10 provinces and 118 districts. According to the 2010 census, there were 4.2 million children aged 7 to 18 years in the country, representing approximately 23% of Zambia’s total population (18 million). The Zambian Educational Statistical Bulletin 2018 was referred to understand how many primary and secondary schools are located in a province and the number of children to aid in assessing potential scaling-up. (Table 7) Provinces located in the central region have a higher child population when compared to the east and west regions (Copperbelt, Central, and Lusaka), where more than 500,000 children live in each of these provinces.

**Table 7:** National and provincial school profiles

	Land area, km^**2**^	No. of districts	No. of schools (children)		Total schools (total children)
			Primary (G1–7)	Secondary (G8–12)	
**National level**					
	752,612	118	9050 (3,339,245)	1117 (861,352)	**10,167 (4,200,597)**
**Province level**					
Copperbelt	31,328	11	990 (423,551)	136 (171,925)	**1126 (595,476)**
Central	94,394	12	1171 (429,995)	124 (102,259)	**1295 (532,254)**
Lusaka	21,896	7	788 (389,162)	122 (136,540)	**910 (525,702)**
Southern	85,283	13	1135 (399,064)	109 (92,952)	**1244 (492,016)**
Eastern	51,476	14	1020 (372,920)	125 (68,595)	**1145 (441,515)**
Northern	77,650	12	887 (312,227)	136 (53,349)	**1023 (365,576)**
Western	126,386	16	978 (268,170)	61 (60,772)	**1039 (328,942)**
North-Western	125,826	11	750 (249,546)	134 (79,297)	**884 (328,843)**
Luapula	50,567	12	643 (272,476)	80 (56,131)	**723 (328,607)**
Muchinga	87,806	10	688 (222,134)	90 (39,532)	**778 (261,666)**

When referring to provincial statistics, a detailed breakdown of the number of schools and children at the district level is needed to facilitate effective planning at the initial stage. Table 8 shows the school profiles in Lusaka province, the smallest region in Zambia but with a high population density.

**Table 8:** District school profiles (Lusaka province)

District level					
Districts	Distance from Lusaka	No. of Primary schools	No. of Secondary schools	Total no. of children	Total no. of teachers
Lusaka	–	668	72	**258,661**	8341
Kafue^a^	51 km	55	24	**36,923**	1002
Chongwe	41 km	89	19	**33,710**	1218
Rufunsa	158 km	65	13	**30,639**	410
Chilanga	26 km	85	23	**25,163**	1142
Chirundu	144 km	45	8	**12,475**	392
Luangwa	315 km	22	7	**11,965**	399

^a^site of the pilot project

When reflecting on the achievement and insights gained from the pilot project, where 154 schoolteachers were trained to screen 18,713 schoolchildren in 73 schools over 6 months, it will be more realistic to gradually expand the SEHP to a new district rather than a province without compromising the essential components in the programme. This is based on the needs and the existing financial and human resources available within the user organisations.

In terms of financial budget, the cost to screen and/or treat a child (with eye drops/spectacles) was estimated to be £3.35, based on the findings from the pilot (URE rate: 3.35%; allergic conjunctivitis rate: 17.0%). This covered the start-up and recurring costs: training workshop for health personnel, consumables, expenditure for MEHCs, transportation subsidy for some families, equipment, medications, and spectacles. By removing the start-up cost, such as the expenses of collaborative meetings and the consultant fees, the cost to screen and treat a child can be reduced by 37.3% (£2.10 versus £3.35). With this costing formula, an estimated £113,000 is required to screen 33,710 children and 1218 schoolteachers in the Chongwe district. (Online Supplementary File 3.)

When introducing SEHP to a new site, careful assessment of the local context is crucial to ensure better accommodation and embrace diversity in cultures. Adaptation of SEHP to local needs and settings cannot be neglected as this determines the level of receptivity among the target populations, which can directly influence the programme’s impact. There are different demographic characteristics and socioeconomic statuses within a country, and some may have cultural beliefs that are distinct from other groups of the population. Despite some inevitable adjustments and adaptations of the programme during scaling up, the essential component will remain intact as they are all inter-connected.

## Discussion

This scalability assessment shows that the feasibility of scaling up the comprehensive SEHP is possible if sufficient funding is available, with great advocacy effort to integrate the eye component into the existing SHNP. Upscaling should be adapted to the local context to ensure all the programme components are intact. Besides, a few strategies and recommendations made as an output of the assessment could facilitate the upscaling process in predicting unwanted shortcomings while improving the programme’s effectiveness and efficacy.

Based on the findings from our assessment, we found that most of the evidence exists to support the potential to upscale and integrate child eye health into SHNP. This included the high competency level of local health personnel such as ophthalmologists, OCOs, ONs, and OTs; the high burden of childhood eye problems, which demonstrated the demand for services; the high level of receptivity shown by the local community such as schoolteachers, school heads, caretakers and children seeking health services; and the potential of cost-saving by sharing resources between the two programmes through incorporating eye health as an additional component to the existing programme. Such success has been highlighted in Ghana and Cambodia [23].

**Fig. 1:**
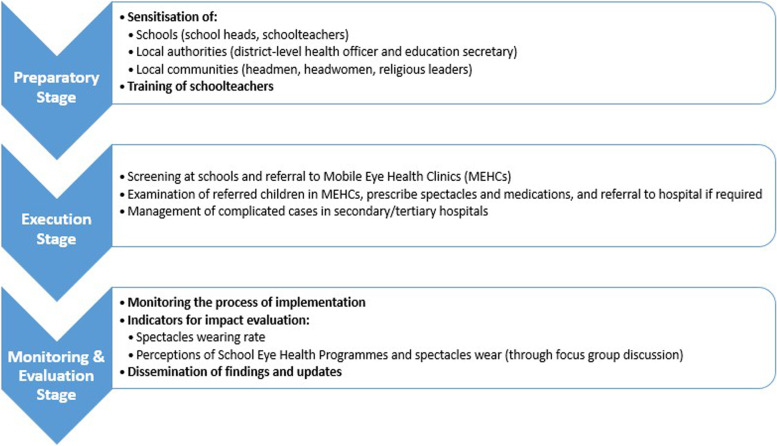
Components and stages of the comprehensive School-based Eye Health Programme

The credibility of the components embedded in the innovation is important to ensure programme’s effectiveness, receptivity from the locals, and achieving maximum health gain [24]. The assessment of the components adopted in the piloted SEHP revealed that all are essential for an effective wide-scale expansion. It consisted of a package of interventions at different stages, each contributing to the programme’s effectiveness. By consolidating the findings from the assessment, we proposed the following model for upscaling. (Fig. 1) Based on the inhibiting and facilitating factors, we recommended that the resource team adhere to this model and ensure that the components are intact when expanding to new sites to reach more Zambian children.

Integration of health programmes in mainstream school health has numerous benefits, such as improving a child’s health while being cost-effective [25]. The Enhanced School Health Programme (ESHI) conducted in Ethiopia incorporated three components: Home Grown School Feeding (HGSF), Water, Sanitation and Hygiene (WASH), and deworming interventions. Results show that only a 25% increment cost for including additional two health interventions in the existing programme refers to US$0.16 versus US$0.20 per child per day [25]. Another costing analysis of the vertical and integrated eye health programme model in Zanzibar revealed that the former utilised 1.2 times more resources than the integrated model [22]. This evidence can be used to advocate for the Zambian government to include the child eye component into the existing SHNP.

While there is sufficient evidence that including prevention and promotion of eye health into a holistic health care system can improve child eye health, [26] some pain points may prevent it from succeeding. The lack of local political commitment remains the most critical barrier, mainly due to health priority and funding shortages [27]. While external funding is important and useful for the initial start-up programme, it is usually uncertain and short-term, as emphasised in the guideline for setting up a sustainable school eye health programme [28]. The ceasing of funding and subsequent disruption of programme continuation possess a risk of losing the locals’ trust, followed by a minimal commitment by the health personnel. Sustainability is key to ensuring programme continuation and, therefore, positively impacting population health. Models such as advocacy to include eye health into the health budget should be explored, especially in a low resource setting like Zambia.

The strength of this assessment is that the pilot implemented in the Kafue district provided insights and constructive lessons learnt for the scaling-up. The pilot has laid a sound foundation and contributed massively to our knowledge in the future upscaling specified to Zambia’s context: Firstly, the model of the comprehensive SEHP that we have adopted is likely to bring a more significant programme impact. Although a few components were added, such as community sensitisation, which may require more time and resources, they are essential to gain community trust and support. Secondly, it is highly confident that the resource team, consisting of highly competent and committed individuals across the three implementing organisations, can lead the programme and mobilise the resources during the scaling up. Thirdly, the networking which has already been established during the pilot will facilitate future work.

Limitations must also be acknowledged. Due to the COVID-19 pandemic, onsite observation and interviews with the local communities, such as the schoolteachers and caretakers, were unable to be conducted. Therefore, the process and outcome evaluation were based on the working group’s pilot report. To reduce reporting bias, we conducted key informant interviews and developed questionnaires to validate data and explore gaps in the pilot project.

## Conclusion

Findings from the assessment suggested that the scaling-up of the comprehensive SEHP is feasible, provided that the ownership is being transferred to the local stakeholders through integrating SEHP into the existing SHNP. This formal institutionalisation secures government funding, and therefore programme sustainability can be ascertained. Continued efforts such as constant engagement with the government and working closely with strong advocates within the government departments will contribute to the integration’s success.

## Supplementary Information

**Additional file 1.**

**Additional file 2.**

**Additional file 3.**

## Data Availability

The data collection tool (questionnaires) used and analysed during the current study are available from the corresponding author on reasonable request.

## Abbreviations

DEBS: District Education Board Secretary
DHO: District Health Officer
IAPB: International Agency for the Prevention of Blindness
MEHC: Mobile Eye Health Clinic
MoGE: Ministry of General Education
MOH: Ministry of Health
NGO: Non-governmental Organisation
OCO: Ophthalmic Clinical Officer
ON: Ophthalmic Nurse
OT: Optometry Technologist
SDG: Sustainable Development Goal
SHNP: School Health and Nutrition Programme
SEHP: School-based Eye Health Programme
UCVA: Uncorrected Visual Acuity
UK: United Kingdom
UN: United Nations
URE: Uncorrected Refractive Error
UTH: University Teaching Hospital
VA: Visual Acuity
VAO: Vision Aid Overseas
VI: Vision Impairment
WHO: World Health Organisations
