# Supplementary material for: Can we scale up a comprehensive school-based eye health programme in Zambia?
Source: BMC Health Serv Res. 2022 Jul 25;22:945. doi: 10.1186/s12913-022-08350-2 (PMC9310673; doi:10.1186/s12913-022-08350-2)
Supplement: Supplementary file 1 — Additional file 1. [file 12913_2022_8350_MOESM1_ESM.docx]

## Data Collection Tool 1

## Questionnaires for VAO – Zambia Team

First of all, we would like to thank you for your willingness to contribute your input for this scalability assessment of a school eye health programme in Zambia. We believed that your experiences working on the ground and also engaging with high-level authorities will give us useful insights in assessing and planning for the scale-up.

A systematic assessment of our existing capacities and resources is key to ensure an effective, efficient and sustainable upscale effort. Realising our strengths and weaknesses will better equip ourselves with necessary skills and leadership that could avoid undesirable shortcomings. Based on the findings, we will also develop a set of constructive mechanisms and strategies.

There are six elements that we would like to seek your opinions based on the experiences that you have been gaining in the pilot project, as following:

**Element 1:**

Components of the comprehensive School Eye Health Programme

**Element 2:**

Implementing organisations/institutions (VAO, MOH, MOE): relationships, capacities, resources, changes

**Element 3:**

Environment

**Element 4:**

Resource team

**Element 5:**

Vertical upscale - Institutionised School Eye Health Programme through National Health Plan/Policies

*(will share in different set of doc)*

**Element 6:**

Horizontal upscale - Expansion of School Eye Health Programme

*(will share in different set of doc)*

*Please note: You might have no answers/unable to decide/uncertainties in some questions, it is fine to indicate those in the provided columns. These will serve as an area that we can consult and reflect together, and include them in our to-do list prior to scale-up.*

| **Abbreviations**  MEHC – Mobile Eye Health Clinic  MOH – Ministry of Health  MOE – Ministry of Education  OCO – Ophthalmic Clinical Officer  ON – Opthalmic Nurse  OT – Optometry Technologist  SEHP – School Eye Health Programme  VAO – Vision Aid Overseas |
| --- |

# Element 1: Components of the comprehensive School Eye Health Programme

1. In your experience from the pilot programme, which components (as shown below) do you think are **central to the success** of a school eye health programme (SEHP)? Please describe your experience on those components and provide evidence if possible. *(may have more than one components)*

| **Screening** | **Referral** | **Treatment** | **Service delivery** |
| --- | --- | --- | --- |
| **Health promotion** | **Training of school teachers** | **Engagement with local authorities/communities** | **Others: *please specify*** |

| **Description of experience and evidence:** |
| --- |

# Element 2: Implementing organisations/institutions (VAO, Zambian MOH and MOE) – relationships, capacities, resources, changes

1. Which will be the lead organisation that will take the overall responsibility for managing the SEHP during upscaling? What are the relationships among Zambian MOH, MOE and VAO?

|  |
| --- |

2. If the division of roles and responsibilities among them has not been determined, how will you achieve that?

|  |
| --- |

If the division of roles and responsibilities among them has been determined, briefly describe below:

|  | **VAO** | **Zambian MOH** | **Zambian MOE** |
| --- | --- | --- | --- |
| Roles and responsibilities |  |  |  |

3. Within the Zambian MOH, MOE and VAO, is there likely to be any opposition (inhibiting factors) to the SEHP. If so, where and at what level? Please describe.

| VAO:  MOH:  MOE: |
| --- |

4. Did the MOH, MOE and VAO have the capacity to implement the comprehensive SEHP that have been tested in the **pilot project**, in term of:

*(Please state YES or NO. If NO, please explain in the Comments how can this gap be filled in future scale up)*

| **Capacity descriptions** | **VAO** | **MOH** | **MOE** | **Comments** |
| --- | --- | --- | --- | --- |
| (i) The policy and legal framework necessary to introduce the comprehensive SEHP |  |  |  |  |
| (ii) Training capacity for:  (a) school teachers  (b) OCOs, ONs, OTs |  |  |  |  |
|  |  |  |  |  |
|  |  |  |  |  |
| (iii) Capacity engaging with:  (a) schools  (b) local leaders/communities |  |  |  |  |
|  |  |  |  |  |
|  |  |  |  |  |
| (iv) Technical skills/quality of care |  |  |  |  |
| (v) Leadership, management/supervision  (was there a coordinator/manager/team leading the project) |  |  |  |  |
| (vi) Personnel to take on the new tasks implied by SEHP  (was there allocation of personnel/new recruitments to work for the project?) |  |  |  |  |
| (vii) Resources:  (a) human resources  (b) financial resources |  |  |  |  |
|  |  |  |  |  |
|  |  |  |  |  |
| (viii) Logistics/supplies:  (a) logistics  (b) supplies: spectacles, medications  (c) equipment: eye examination instruments  (d) others |  |  |  |  |
|  |  |  |  |  |
|  |  |  |  |  |
|  |  |  |  |  |
|  |  |  |  |  |
| (ix) Physical facilities:  (a) for screening  (b) for referral (MEHC)  (c) secondary/tertiary eye hospitals  (that can manage complex cases) |  |  |  |  |
|  |  |  |  |  |
|  |  |  |  |  |
|  |  |  |  |  |
| (x) Values and orientation supportive of the SEHP |  |  |  |  |
| (xi) Monitoring and evaluation capacity:  (a) data collection  (b) data management and analysis  (c) evaluation |  |  |  |  |
|  |  |  |  |  |
|  |  |  |  |  |
|  |  |  |  |  |

5. Besides working closely with MOH and MOE, do you think by engaging and collaborating with other institutions/organisations in scale-up will yield greater results? If yes, please explain and describe your plan. (e.g. CBM, OEU, SSI, Save the Children, etc.)

|  |
| --- |

6. Are there impending changes within VAO, MOH and MOE that might affect the success of scaling up?

*(Please state YES or NO. If YES, please explain in the Comments how can this be addressed in future scale up)*

| **Impending changes** | **VAO** | **MOH** | **MOE** | **Remark** |
| --- | --- | --- | --- | --- |
| (i) Personnel changes (e.g. Country director, programme manager, officers, representatives, coordinators, etc.) |  |  |  |  |
| (ii) Funding  (a) for operational (training for teachers, pay to trainers, administration, monitoring and evaluation, MEHCs, etc.)  (b) for supplies (medicines, spectacles, disposables, etc.) |  |  |  |  |
|  |  |  |  |  |
|  |  |  |  |  |
| (iii) Health sector reform |  |  |  |  |
| (iv) Others |  |  |  |  |

# Element 3: Environment

1. Learning from the pilot project, did the local people demonstrate the need for child eye health services? What have you observed? Please share your experiences.

|  |
| --- |

2. Did you encounter any challenges/opportunities when working with various stakeholders during the pilot project? Do you think these challenges/opportunities have an impact on the upscale? Please describe.

| **Stakeholders** | **Challenges** | **Opportunities** |
| --- | --- | --- |
| MOH |  |  |
| MOE |  |  |
| Local authorities/communities |  |  |
| School teachers/heads |  |  |
| School-going children  (during screening, follow-up) |  |  |
| Parents |  |  |
| Others |  |  |

3. If the optical supply was to be imported to Zambia, is there any tax exemption from the government? e.g. spectacles (frames and lenses), medications.

If not, are there any approaches that needed to be taken so that the cost/expenses can be minimized.

|  |
| --- |

4. In terms of logistics, were the locations set up for MEHCs accessible by local people? If not, what strategies are you considering/taking to mitigate the lack of access?

|  |
| --- |

#

# Element 4: Resource team

The table below shows the construction of a resource team for a SEHP (data extracted from the protocol).

*Please insert your input into the table (if there is any); please also edit the information if there are any corrections.*

|  | Stakeholders/Personnel | Responsibilities/Roles |
| --- | --- | --- |
| National/Country level | VAO country team  - Country Director  - Programme officer/s  - Part-time: master trainers, consultant | - Supervision: supported the logistic of the survey. - Survey management team: ensure proper data collection, storage and analysis at each stage of survey implementation. (with MOH and MOE) - Supported logistic training, MEHC, quality assurance, monitoring and evaluation. - Provided additional personnel. - Visited schools and communities (with the accompaniment of DHO and DEBS) |
|  | MOH representatives  - National Eye Health Coordinator | - Supervision: supervised the entire SEHP. - Survey management team: ensure proper data collection, storage and analysis at each stage of survey implementation. (with VAO and MOE) - Organised session for information dissemination among stakeholders. (with the support from VAO) |
|  | MOE representatives | - Supervision: supervised the entire SEHP. - Survey management team: ensure proper data collection, storage and analysis at each stage of survey implementation. (with MOH and VAO) - Organised session for information dissemination among stakeholders. (with the support from VAO) |
| Province level | Provincial Health Director | - Selected eye health personnel and teachers to be trained for the programme. |
|  | Provincial Education Officer | - Selected eye health personnel and teachers to be trained for the programme. |
| District level | District Health Officer  (DHO) | - Accompanied VAO team to visit schools and invite for participation. - Accompanied VAO team to visit local communities. |
|  | District Education Board Secretary  (DEBS) | - Accompanied VAO team to visit schools and invite for participation. - Accompanied VAO team to visit local communities. |
| School level | School Heads | - Engaged with VAO, DHO and DEBS, and provided support to SEHP. |
|  | Head Teachers | - Contacted parents/guardians of children with eye problems. School teachers reported to Head Teacher. - Will collect data (spectacle wearing compliance) from school teachers and submit to DEBS – *interrupted due to Covid-19* |
|  | School management officers | - Trained as screeners. |
|  | School teachers  (those in-charged of School Health and Nutrition programme) | - Trained as screeners. - Will observe spectacle wearing compliance and report to Head teacher – *interrupted due to Covid-19* |
| Eye health personnel /  Mobile eye health clinic | Ophthalmic Nurses (ONs)  Ophthalmic Clinical Officers (OCOs)  Optometry Technologists (OTs) | - Follow-up referred children in MEHC: refraction and dispensed spectacles, prescribed medications for conjunctivitis, referral to tertiary care. - Trained school teachers as screeners. |
|  | Ophthalmologists  Optometrists | - Provided tertiary care in hospitals. - Trained school teachers as screeners. |
|  | Focal person for MEHC | - Spent at least one day per fortnight in the field with each team to provide on-site mentorship and supportive supervision of diagnostic accuracy, as well as other technical, logistical and moral support as required. - Reported to District Health Director and DEBS |
|  | *Others?* |  |

1. Will the roles and responsibilities for each stakeholder as described above change during upscaling? Please specify.

|  |
| --- |

2. Who facilitated the development and testing of the pilot programme? Please list the individual/s accordingly.

|  |
| --- |

3. Will these individuals be part of the resource team during upscaling? If not, what approaches that might have to be considered to ensure no disruption during the scale-up?

|  |
| --- |

4. Have additional individuals been identified as additional members of the resource team to assist in upscaling? If yes, please describe their role and responsibilities.

|  |
| --- |

4. *Leadership, management, resource mobilization, strategies development, dissemination and advocacy, monitoring and evaluation, research, etc.*

Besides commitment, skills listed above are essential for a resource team to effectively upscale the SEHP. Is there a need for additional training or skills development for resource team? If yes, please specify for whom and which areas need to be strengthened.

|  |
| --- |

5. Are the financial resources available/sufficient to support the resource team? (e.g. upscale to at least two more districts) If not, what plans do you have in place?

|  |
| --- |

6. Are there any mechanisms to retain key members? (key members: individuals leading the programme)

|  |
| --- |

# Element 5: Vertical upscale - Institutionalised School Eye Health Programme through National Health Plan/School Health and Nutrition Programme

**Vertical upscale through advocating for the inclusion of School Eye Health Programme (SEHP) in the National Health Plan/School Health and Nutrition Programme is crucial to ensure full formal support from the government. This is especially referring to resources allocation such as manpower and funding (national health budget). Effective leadership also plays a key role in determining the success of the programme.**

Eye health has been included as one of the non-communicable diseases that needed to be prioritised in the National Health Strategic Plan 2017-2021 (NHSP) (1). Besides, in the 3^rd^ National Eye Health Strategic Plan (NEHSP), it aimed to achieve eye health coverage across the country to at least 90% by the year 2021 (2). SEHP, an intervention that is likely to reach maximum children coverage in communities, can be one of the effective approaches to achieve this goal. Below are the statements stated in the NHSP 2017-2021 for eye health services focusing in schools and communities (page 47) (2):

4.7.2 Eye Health Services


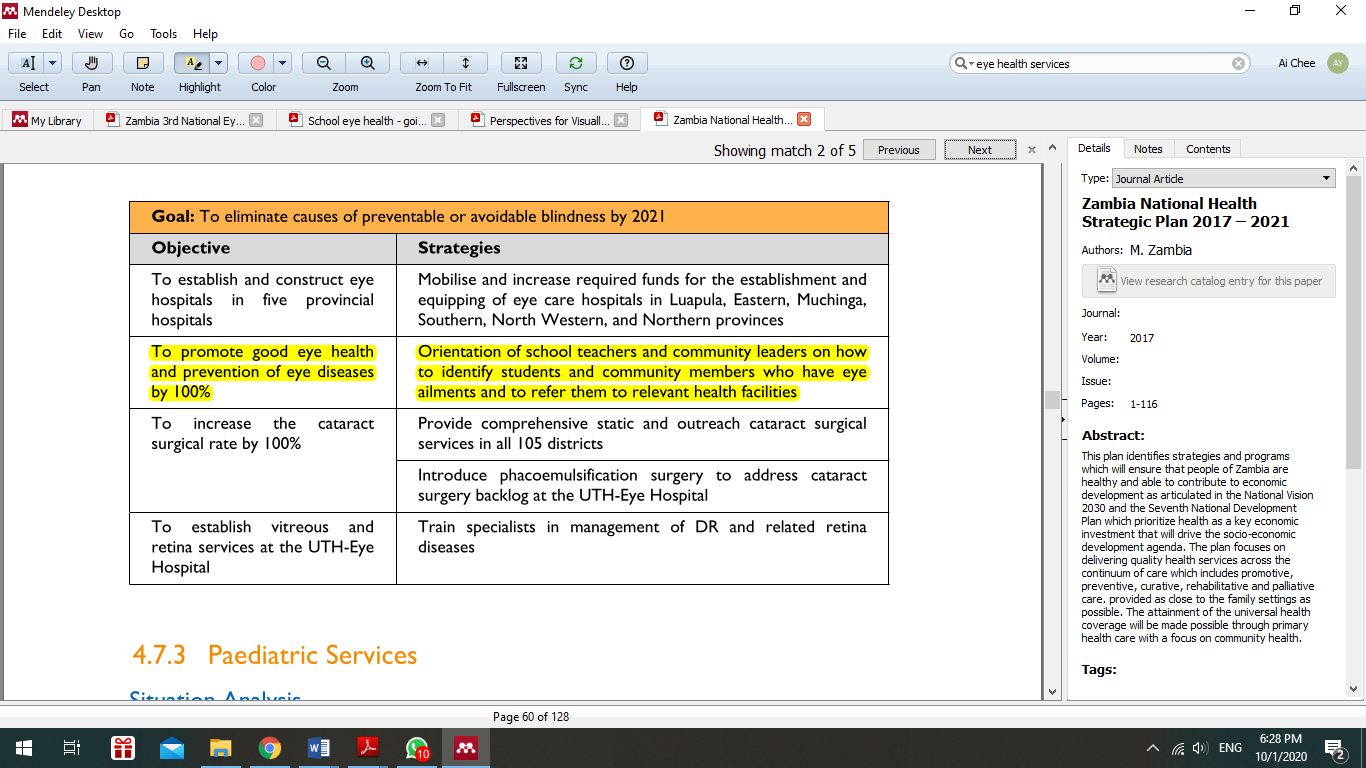


1. Despite the strategy of capacity building among school teachers and community leaders in detecting common eye diseases was mentioned in the NHSP 2017-2021, a comprehensive description of the **SEHP** was not highlighted. With this, what changes are needed at the policy level to ensure that the scale-up can proceed and is sustainable?

|  |
| --- |

2. How can you achieve these changes as mentioned above?

|  |
| --- |

3. Who will advocate for these changes?

|  |
| --- |

4. Are there advocates within MOH and MOE that will support such changes? Or are there other stakeholders (organisations/institutions) that had experience in this area that you can engage with?

If yes, detail your plan working with them.

|  |
| --- |

5. By when can you realistically expect these changes to be accomplished/reaching to another milestone? Please provide a brief timeline.

|  |
| --- |

6. Are there mechanisms in place for monitoring and evaluating the advocacy progress as the scaling-up proceed?

|  |
| --- |

# Element 6: Horizontal upscale - Expansion of School Eye Health Programme

**The goal of replicating/extending the implementation of SEHP to other geographical sites is that the impact of the programme could benefit larger or different populations.**

Collaborating with Zambian MOH and MOE in the planning and implementation of piloted SEHP in Kafue district is one of the strengths that should be highlighted. Working with government ministries have contributed greatly to building local ownership. For instance, local resources from the public sector were utilized (ophthalmologist in a tertiary hospital, OCOs and ONs to train school teachers, province and district officers to visit local communities and schools).

1. Will the learning and outcomes from the pilot project help you in scaling-up the SEHP to other districts? How will they help?

|  |
| --- |

2. When expanding the SEHP to a **new district**, there are new stakeholders that we need to engage; existing stakeholders that we need to strengthen vision. The table below presenting stakeholders at various levels with their respective role in SEHP.

What needs to be done to transfer the knowledge/communicate with them before/during/after the scale-up. By completing the table below, you will visualize steps of engagement and communication. (suggestion: in some circumstances, you might want to hold a collaborative meeting with existing stakeholders to refresh the aim of the SEHP, or allocation of task and responsibilities.)

Important: Please note that your message/communication with stakeholders should be clear and understandable.

|  | **Key stakeholder** | **N/E*** | **Key roles in SEHP** | **What needs to be done?** |
| --- | --- | --- | --- | --- |
| **Ministry of Health** | National eye health coordinator | E | - Supporting and mobilizing resources (national level). - Supervision of the entire SEHP. |  |
|  | District health officer | N | - Supporting and mobilizing resources (district level). - Visiting all the schools and communities. |  |
|  | Senior Medical Superintendents of Eye Hospitals/Clinics |  | - Supporting referrals from MEHCs. |  |
|  | Ophthalmologists |  | - Supervising OCOs and ONs during MEHCs. - Getting referrals from MEHCs. |  |
|  | Optometry Technologists |  | - Getting referrals from MEHCs. |  |
|  | OCOs, ONs, OTs | N | - Training school teachers. - Conducting MEHCs. - Referring children. |  |
| **Ministry of Education** | District education board secretary (DEBS) | N | - Supporting and mobilizing resources (district level). - Visiting all the schools and communities. |  |
|  | Guidance teacher and planning officer | N | - Supporting logistic by guiding trip to schools. |  |
|  | School heads/head teachers | N | - Supporting and mobilizing resources (school level). |  |
|  | School teachers | N | - Conducting eye health screening (school level). - Monitoring children spectacles wearing rate. |  |
| **VAO** | Country director | E | - Supervision of the entire SEHP. - Supporting and mobilizing resources (VAO). - Monitoring and evaluation of the entire SEHP. - Generating evidence-based research |  |
|  | Programme and Project officer | E | - Supervision of the entire SEHP. - Providing technical support. - Mobilizing resources. - Visiting all the schools and communities. - Monitoring and evaluation of the entire SEHP. - Generating evidence-based research |  |
| **Local Community** | Local leaders | N | - Supporting and are aware of the SEHP. |  |
|  | Local communities | N | - Supporting and are aware of the SEHP. |  |
|  | Parents | N | - Supporting and are aware of the SEHP. |  |
|  | Others  *(please specify)* |  |  |  |
| * N = New stakeholder; E = Existing stakeholder | | | | |

3. The capacity of implementing organisations (MOH, MOE and VAO) plays a critical role in determining the magnitude of the scale-up (how many districts/provinces that we are going to implement SEHP). Below shown the resources used in the pilot project.

School eye health pilot project in Kafue district from June-December 2019 (6-month) (3):

- covered 73 schools (Grade 1 to 9)
- screened 18,713 children (out of 43,370 children, coverage of 43.15%)
- trained 154 school teachers
- engaged with 3 optometry technologists and 1 ophthalmologist
- utilised 3 teams of OCOs and ONs for MEHCs in 6 zones (1 OCO and 1 ON formed a team serving 2 zones)
- time frame:

Phase I 🡪 training of teachers – 17^th^ June to 31^st^ July (1.5 months)

Phase II 🡪 management of referrals in MEHCs – 29^th^ September to 17^th^ December (2.5 months)

- outcome: identified 3817 children with eye problems (600 spectacles dispensed, 68 referrals to a tertiary hospital, 3000 were prescribed eye drops)
- total expenditure: estimated £60,000 (fully supported by VAO, while MOH paid the salary of health personnel)

Based on the reflection above, and your familiarity of geographical areas/resources in Zambia, which **three districts** would you propose for our future scale-up? Which **one province** would you propose for the next SEHP? Please explain those contributing factors.

*(before naming those districts/province, you may want to refer to the literature/resources outlined below - geographical and schools profile, human resources, funding)*

|  |
| --- |

**BACKGROUND – GEOGRAPHICAL & SCHOOLS PROFILE**


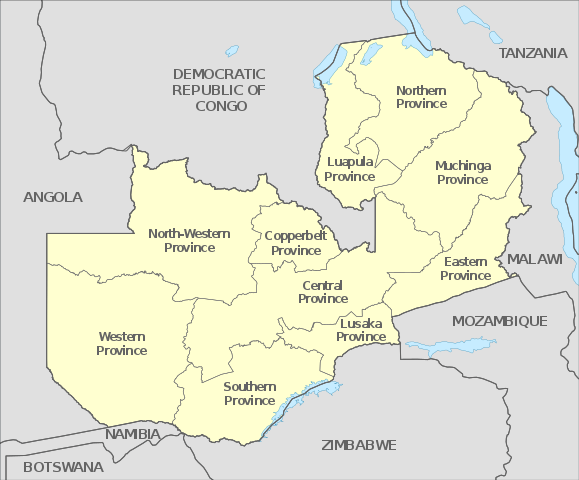

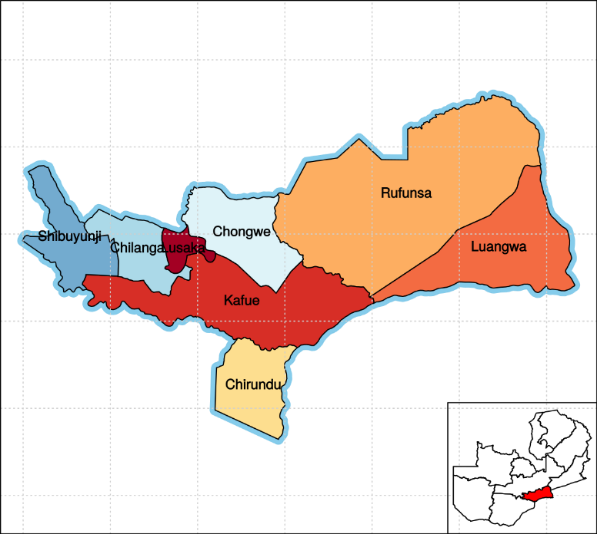


Zambia has 10 provinces and 118 districts.

Table below shows the geographical areas, number of districts, schools and children by referring to Zambia Educational Statistical Bulletin at province level (updated in 2018) (4):

|  | **Land area, km^2^** | **No. of districts** | **No. of schools (children)** | | **Total** |
| --- | --- | --- | --- | --- | --- |
|  |  |  | **Primary (G1-7)** | **Secondary (G8-12)** |  |
| **National level** | | | | | |
|  | 752,612 | 118 | 9,050 (3,339,245) | 1,117 (861,352) | **10,167 (4,200,597)** |
| **Province level** | | | | | |
| Copperbelt | 31,328 | 11 | 990 (423,551) | 136 (171,925) | **1,126 (595,476)** |
| Central | 94,394 | 12 | 1,171 (429,995) | 124 (102,259) | **1,295 (532,254)** |
| Lusaka | 21,896 | 7 | 788 (389,162) | 122 (136,540) | **910 (525,702)** |
| Southern | 85,283 | 13 | 1,135 (399,064) | 109 (92,952) | **1,244 (492,016)** |
| Eastern | 51,476 | 14 | 1,020 (372,920) | 125 (68,595) | **1,145 (441,515)** |
| Northern | 77,650 | 12 | 887 (312,227) | 136 (53,349) | **1,023 (365,576)** |
| Western | 126,386 | 16 | 978 (268,170) | 61 (60,772) | **1,039 (328,942)** |
| North-Western | 125,826 | 11 | 750 (249,546) | 134 (79,297) | **884 (328,843)** |
| Luapula | 50,567 | 12 | 643 (272,476) | 80 (56,131) | **723 (328,607)** |
| Muchinga | 87,806 | 10 | 688 (222,134) | 90 (39,532) | **778 (261,666)** |

🡪 **Copperbelt** province had the highest number of children, followed by **Central, Lusaka, Southern** and so on.

🡪 Lusaka had the smallest land area.

Table below shows the school profiles in Lusaka province (information from MoGE updated in 2019):

| **District level** | | | | | |
| --- | --- | --- | --- | --- | --- |
| **Districts** | **Distance from Lusaka** | **No. of Primary schools** | **No. of Secondary schools** | **Total no. of children** | **Total no. of teachers** |
| Lusaka | - | 668 | 72 | **258,661** | 8,341 |
| Kafue | 51 km | 55 | 24 | **36,923** | 1,002 |
| Chongwe | 41 km | 89 | 19 | **33,710** | 1,218 |
| Rufunsa | 158 km | 65 | 13 | **30,639** | 410 |
| Chilanga | 26 km | 85 | 23 | **25,163** | 1,142 |
| Chirunda | 144 km | 45 | 8 | **12,475** | 392 |
| Luangwa | 315 km | 22 | 7 | **11,965** | 399 |

Highlighted in yellow: area and school profile of our pilot project.

🡪 **Lusaka district** had the highest number of children, followed by **Kafue, Chongwe**, and the following as the above table shown in chronological order.

Age distribution in Zambia’s education system (5):

| **Level** | **Grade** | **Age distributions** |
| --- | --- | --- |
| Early childhood education | **-** | 3 to 6 years old |
| Primary | 1 to 7 | 7 to 13 years old |
| Secondary | 8 to 12 | 14 to 18 years old |
| Tertiary | Universities/ Colleges | 19 years old and above |

* For our scale-up, we will only screen and examine children in grade 1 to 12 (age 7 to 18 years).

**EXISTING HUMAN RESOURCES**

There was an assessment of eye health system in year 2011. Table below shows the distribution of eye health workers at the province level (6):


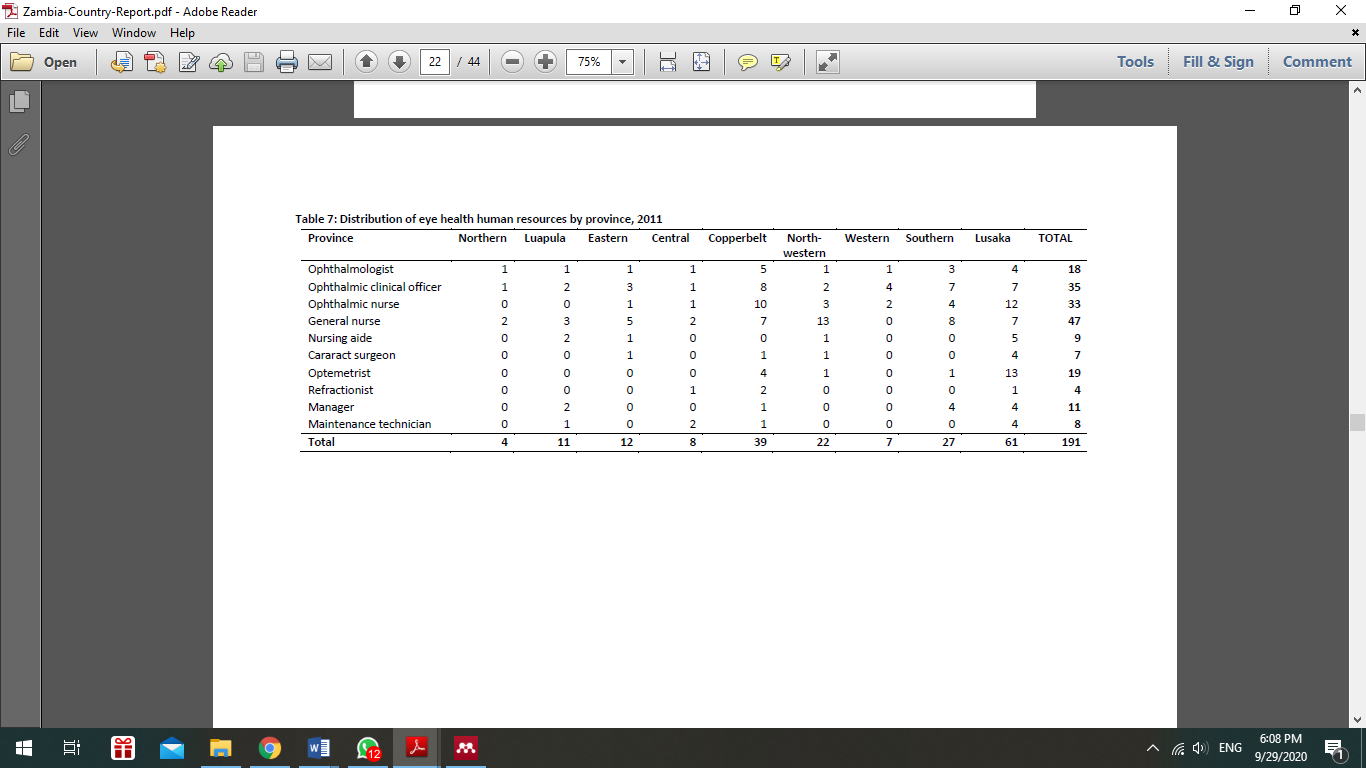


🡪 Lusaka province had the highest number of eye health personnel, followed by Copperbelt and Southern province.

Since the data above was captured in 2011, there is a higher possibility that the data might have outdated. Therefore, we refer to the National Eye Health Strategic Plan 2017-2021 on the distribution of eye health workers, as below (1):

|  | **Northern** | **Luapula** | **Eastern** | **Central** | **Copperbelt** | **North-Western** | **Western** | **Southern** | **Lusaka** | **Muchinga** | **Total** |
| --- | --- | --- | --- | --- | --- | --- | --- | --- | --- | --- | --- |
| **Ophthalmologist** | 0 | 1 | 1 | 2 | 7 | 1 | 1 | 0 | 9 | 0 | **24** |
| **Optometrist** |  |  |  |  |  |  |  |  |  |  | **2** |
| **Ophthalmic Clinical Officer (OCO)** |  |  |  |  |  |  |  |  |  |  | **49** |
| **Ophthalmic Nurse (ON)** |  |  |  |  |  |  |  |  |  |  | **88** |
| **Optometry Technologist (OT)** |  |  |  |  |  |  |  |  |  |  | **34** |

🡪 Due to limitation in data search, only distribution of ophthalmologist at province level was presented.

🡪 Consistent with the assessment in 2011, Lusaka and Copperbelt province had the highest number of ophthalmologists.

🡪 The number of ONs and OCOs are higher among the other workforces (88 and 49 respectively).

**BUDGET (Funding)**

Reflected from the pilot project’s actual expenses, the cost to screen or follow-up a child was estimated. (table below) You can enter the total number of children that you anticipated to cover in a new district/province (column B) and multiply the cost per child (column A). This will generate the average funding that is required for the scale-up (column C).

| **Items** | **Estimated Cost, £/child**  **(A)** | **Number of children**  **(B)** | **Total**  **(C)** |
| --- | --- | --- | --- |
| To screen and provide management (e.g. spectacles/medications/referral) | £3.50* |  | **£** |

* £60,000 divided by 18,000 children (18,000 were screened and among them, 6,000 were provided with either spectacles or medications).

Please note: During scaling-up, the cost to screen and provide management for a child might be lower than the pilot, as there is less likely to purchase new sets of equipment and instruments.

4. Do you think that the vertical (integration of SEHP into SHNP) and horizontal (expansion) scaling-up can happen at the same time? If yes, what are your strategies; If no, what are the challenges and how can you address that?

|  |
| --- |

5. The following are key factors that need to be monitored in the progress of expansion:

- Whether all the components of the comprehensive SEHP are implemented
- Whether local adaptation maintains the essential elements of the comprehensive SEHP
- Whether local adaptation continues to produce the desired outcomes and impact of the comprehensive SEHP
- Impact on overall service delivery and quality of care
- Ongoing environment changes
- Unexpected results (e.g. community empowerment, provider sense of confidence), with either positive or negative impact
- Perceptions of progress and problems at each level of service delivery

Which of these can be monitored and evaluated using the existing management and information system or other existing sources of information?

|  |
| --- |

6. What new systems need to be established (or have already been established) for such monitoring? Who will be responsible for monitoring/evaluation and are the resources for such monitoring available? If not, what needs to be done?

|  |
| --- |

# References:

1. Zambia M of HR of. Zambia National Health Strategic Plan 2017 – 2021. 2017;1–116. Available from: https://www.medbox.org/countries/zambia-national-health-strategic-plan-2017-2021/preview%0Ahttp://www.moh.gov.zm/docs/ZambiaNHSP.pdf

2. Muma K, Mumbi W. Eye Health In Zambia. Heal Press Zambia. 2019;03(12):2.

3. School eye health pilot survey in Kafue district. 2020;

4. Education G. Republic of Zambia Ministry of General Education (Bulletin). Bull Educ Stat [Internet]. 2016;(January):70. Available from: http://www.moge.gov.zm/?wpfb_dl=50

5. Zambia Education System [Internet]. [cited 2020 Sep 14]. Available from: https://www.scholaro.com/pro/Countries/Zambia/Education-System

6. Bozzani and Griffiths. Situation analysis of eye health care in Zambia. Repub Zambia Minist Heal Int Cent Eye Heal Sightsavers. 2011;(December):1–44.
