# Supplementary material for: Can we scale up a comprehensive school-based eye health programme in Zambia?
Source: BMC Health Serv Res. 2022 Jul 25;22:945. doi: 10.1186/s12913-022-08350-2 (PMC9310673; doi:10.1186/s12913-022-08350-2)
Supplement: Supplementary file 2 — Additional file 2. [file 12913_2022_8350_MOESM2_ESM.docx]

## **Data Collection Tool** 2

## Stakeholder Interview Question Guide

- For the National Eye Care Coordinator (Representative of the Ministry of Health of Zambia)

**(A) Views on consensus and partnerships**

We know that to successfully scale up the SEHP, each stakeholder is playing their respective roles: the Ministry of Education will engage with school authorities and schoolteachers; the Ministry of Health will mobilise the eye health personnel for teachers’ training and for the mobile eye health clinics; VAO will contribute in coordinating the programme.

Q: What do you think of the roles mentioned just now? Do you think we can further strengthen and expand these roles?

Q: Do you think all the three stakeholders (i.e. the MOH, MoGE and VAO) are reaching a consensus that the ultimate goal is to expand the programme to other districts and work towards formalising it? meaning to make the programme formally adopted by the government so it can be funded and made more sustainable. (do you think all the three stakeholders are on the same page about this vision?)

**(B) Views on the integration**

Q: Now, let’s move on to the second area. In your opinion, how likely do you think the success of the integration of the School eye health programme into the School health and nutrition programme? Can you kindly share from the MOH’s standpoint on this?

Q: Do we have a key advocate in MoGE that can help us advocate for this integration?

Q: Can you then give us some of the facilitating factors that can help to enable further this integration process? or any practical steps that we can work on systematically?

Q: Besides positive sides, are there any potential obstacles that you think could hinder this process and your suggested mitigation strategy?

Q: Based on your experience, how long does it take for a policy to be formalised by the government? Do you have any idea about it?

**(C) Views on the health information system in Zambia**

As we know, health data are important for policy development and guide programme planning and implementation to meet the population's needs. During the pilot project, there were workshops held to build the capacity for data management and analysis due to the lack of capacity within the resource team.

Q: So we have been thinking, is there any possible way the MOH can mobilise its health information system resources to collect and analyse the data in the future upscaling? This is because this approach will save funds and allow the inclusion of child eye health data in the national health management information system (HMIS).

Q: Do you think we can collaborate with the HMIS to manage the upscaling data? Can you share how we can collaborate with them in the programme?

Q: Are there anything that you would like to add?

- For the Kafue District Education Planner (Representative of the Ministry of General Education)

**(A) Views on consensus and partnership**

To successfully scale up the SEHP, each stakeholder's allocation and clarity of roles in mind are important. For instance, MoGE will engage with school authorities and schoolteachers; MOH will be mobilising eye health personnel for schoolteachers’ training and mobile eye health clinics; VAO will contribute to coordinating the programme.

Q: What do you think of the roles laid out above?

Q: How can we further strengthen and expand these roles?

Q: Are all the three stakeholders reaching a consensus that the goal is to expand the programme and work towards institutionalising it? (i.e. the inclusion of SEHP into the SHNP so that it will be a more sustainable approach to providing eye care services to schoolchildren)

**(B) Views on the integration**

Q: In your opinion, how likely do you think the success of the integration of SEHP into the SHNP? Can you kindly share from the MoGE’s standpoint on this?

Q: Can you give us some of the facilitating factors or practical steps that can help to enable this integration process?

Q: Are there any potential obstacles that could hinder this process? Can you suggest any mitigation strategy for that?

Q: Based on your experience, how long does it take for a policy to be formalised?

Q: What do you think about the idea of incorporating eye health education material into the curriculum? What are some of the initial steps that we can take?

Q: Do you have any comments and thoughts that you would like to share with us?
