# Supplementary material for: Can we scale up a comprehensive school-based eye health programme in Zambia?
Source: BMC Health Serv Res. 2022 Jul 25;22:945. doi: 10.1186/s12913-022-08350-2 (PMC9310673; doi:10.1186/s12913-022-08350-2)
Supplement: Supplementary file 3 — Additional file 3. [file 12913_2022_8350_MOESM3_ESM.docx]

Table below represents the demand and needs assessment which can help in planning in terms of considering about the required resources based on the local demand. This framework is supported by referencing to the pilot.

|  |  | **Kafue Pilot Project**  ***(for reference)*** | **Chongwe**  ***(anticipated district for expansion)*** | **Remark** |
| --- | --- | --- | --- | --- |
| **Demand** | | | | |
| No. of children |  | 18,713 | 42,000 | *The size of children population is almost double.* |
| No. of schools |  | 73 | 110 |  |
| **Needs (required)** | | | | |
| No. of school teachers needed to be trained | Human resources | 146 |  |  |
| No. of OCOs and ONs |  | 2 |  |  |
| No. of optometrists |  | 1 |  |  |
| No. of ophthalmologists |  | 1 |  |  |
| No. of children needed follow up  - for MEHC  - for surgery interventions/ ophthalmologist management in hospitals  - no. of children prescribed spectacles  - no. of children treated with eye medications | Referral | 2818  (actual turn up in MEHC: 5958, 3817 had a diagnosis)  68  621  3,184 |  |  |
| Financial resources  - training  - spectacles and medications  - logistics  - monitoring and evaluation | Financial resources | £62,455.27  (£3.35/child) | £ ___  (£3.35/child) |  |
| Timing  - school teachers briefing &  training  - school teachers conduct eye  screening at schools  - ONs, OCOs and OTs conduct  follow-up of referred children  (MEHC) | Timeline | 1.5 months  2.5 months |  |  |

Through completing the above exercises, the practicality to scale up the SEHP in Chongwe district can be determined. Furthermore, it can also be served as a preparatory stage to further equip what is lacking before the scaling-up takes place.
